# Embodied dynamics of creative placemaking: conceptualizing the spaces, places, and environments of creativity

**DOI:** 10.3389/fpsyg.2025.1656337

**Published:** 2025-12-19

**Authors:** Laura H. Malinin

**Affiliations:** Department of Design and Merchandising, College of Health and Human Sciences, Colorado State University, Fort Collins, CO, United States

**Keywords:** embodied creativity, enactive architecture, creative press, creative workplace, behavior setting theory, affordance theory, creative placemaking, 4E cognition

## Abstract

Researchers have long called for an integrated approach toward understanding the complex relationships between factors influencing creativity. Significant efforts have been made in this area through research examining interactions between personal traits, social environments, and creative productivity. However, relationships among factors involving the physical context of creativity have less often been the focus of cross-disciplinary inquiry, due, in part, to lack of any conceptual framework connecting them. This leaves interior designers, architects, and facility planners left to rely largely on anecdotal evidence and fragmented empirical insights to inform creative placemaking efforts through their environmental designs. Recent embodied creativity approaches have argued that to advance creative process knowledge and facilitate creativity in educational and work environments, creativity must be considered a physically and socially situated practice. There is empirical evidence that professionally and historically creative people are aware that the physical environment is an important factor in their creative productivity, that they leverage and manipulate features and qualities of a setting during creative efforts, and attribute insights and productivity to specific settings. Yet there is no framework linking the physical environment and creative processes, suggesting a lack of awareness about how designed environments might be a resource for creativity. This conceptual analysis aims to bridge this gap by utilizing an *embodied creativity* lens, grounded in ecological psychology and 4E (embodied, embedded, enactive, extended) cognitive science, to evaluate and synthesize literature from creativity, environmental design, ecological psychology, and cognitive science towards clarifying person-environment relationships during creativity, including conceptualizations of creative space, place, and physical environments supporting creativity.

## Introduction

Historically, creativity research has largely focused on cognitive processes (typically divergent and convergent thinking) with research shaped by the “standard” definition of creativity as an ability to come up with ideas that are both “novel” and “useful” (Runco and Jaeger, 2012). Recent *embodied creativity* approaches have argued that to advance the field, creativity must be considered as physically and socially situated, best examined as a dynamic system (Glăveanu, 2014; Malinin, 2019; Sawyer and DeZutter, 2009). This perspective aligns with accounts of historical creativity describing attributes of designed and natural environments that people believed were instrumental to their creativity (Buttimer, 1983; Csikszentmihalyi, 1996; Fig, 2009; Malinin, 2016). Furthermore, there is a rich history of building designs intended to foster individual and organizational creativity, often through aesthetic approaches to inspire the imagination or through spatial strategies to foster serendipitous social interactions that spur creative collaboration. Yet efforts to understand how the designed environment factors into creative processes have been limited when compared to other areas of creativity research due, in part, to disciplinary-siloed research approaches complicated by conceptual, methodological, and analytical variety (Blomberg and Kallio, 2022). This conceptual analysis draws upon literature from creativity, environmental design,^1^ ecological psychology, and cognitive science to (a) surface three overarching problems and (b) propose how a 4E (embodied, embedded, enactive, and extended) cognition lens might be used to address them, laying the groundwork for an integrated and interdisciplinary framework.

### 4E cognition

4E cognitive science is an interdisciplinary field of research concerned with the role the body plays in cognition, including how cognition involves neural and extraneural processes and environmental coupling (Gallagher, 2023). Embodied cognition is both an “umbrella” term to describe the field as well as one of the “4Es” (Newen et al., 2018). Even within and between 4E perspectives there are sometimes conflicting or overlapping positions, which can further lead to conceptual confusion. All 4E perspectives generally agree that cognition involves some degree of person-environment coupling, the debate between (and within) the perspectives is whether – and, if so, to what extent – cognition involves mental representation (Chemero, 2009, p. 43; Gallagher, 2015, p. 1). One perspective, referred to as “strong” embodiment or radical embodied cognitive science (RECS), is grounded in concepts tracing back to Gibson’s (1977) ecological psychology and theory of direct perception. RECS rejects computational theories of mind, instead theorizing that action and perception are intertwined, with cognition enacted through body-environment interactions (Thompson and Varela, 2001, p. 418; Gallagher, 2015, 2023). For the purposes of this paper and its focus on the physical designed environment and creativity, the 4Es are defined as follows. *(Strong) embodiment* positions the body as central to cognition; sensory experiences and perception depend upon the anatomy and physiology of the body, including ways the body moves. *Embedded cognition* concerns how a person offloads cognitive work to the physical environment. This perspective seeks to explain how the physical environment constrains, shapes, and enables cognition and understand ways that people manipulate their environments to improve cognitive performance. *Enactive cognition* argues that perception is for action; people think-in-action and perceive their physical environments in terms of functional relevance. *Extended cognition* posits that person-environment coupling extends the cognitive system beyond the body; material artifacts and technologies in the physical environment play a co-constitutive role, as do other people.

### Conceptual analysis: approach and methods

This conceptual analysis seeks to answer two key questions: *What are the barriers to better understanding the role of the designed environment in creativity*? and *How might these barriers be overcome to better understand person-environment relationships during creative endeavors*? These questions are driven, in part, by the author’s lived experiences as a practicing architect, design educator, and cognitive scientist working in the United States. Thus, the scope of this analysis is focused primarily on building architecture and interior design, with illustrative examples of environmental designs mainly within the U.S. *Creative placemaking,* in this context, refers to buildings and interiors designed to support users’ individual and social creativity. This differs in scale from how the term is often used in the literature and popular press, which is to describe city and urban planning efforts to attract and retain creative talent (e.g., Landry, 2012) or to strengthen communities through the integration of arts and cultural activities (e.g., National Endowment for the Arts, n.d.).

To answer Question 1, the author conducted an extensive review of the literature, beginning with broader search terms (e.g., “creativity + physical environment,” “creativity + office design,” “creativity + learning space design”) Articles were selected based on relevance, journal quality, date of publication, impact, methodology, and research quality and analyzed for key themes, including (a) architectural design strategies and evidence of impacts, (b) references to creativity models or theories, and (c) definitions or conceptualizations of creativity, as well as challenges or limitations of the research. This first level analysis guided more focused searches to identify and interrogate three overarching problems in response to Question 1: *Awareness, Definition,* and *Conceptualization*. For example, a deeper investigation into the Awareness problem narrowed search terms to specific qualities or features, such as “creativity + artificial lighting.” Additionally, key articles were used to conduct backward and forward citations searching to identify any important literature previously uncovered. In total, 108 peer-reviewed articles – including 83 original research papers (45 experimental design, 26 case study, and 12 survey/interview methods) and 26 comprehensive reviews – were analyzed for Question 1 and informed three propositions in response to Question 2.

## The awareness^2^ problem: the physical environment’s role in creativity

### Mental and social processes of creativity

Mathematician Jules Henri Poincaré (1910) famously wrote about the moment creative insight struck when he stepped aboard a bus. His story of mathematical creativity describes in detail how he intentionally immersed himself in different environments when trying to solve a difficult problem, whether by walking the streets near his home or by seeking out inspirational settings, such as bluffs along the seaside. Poincaré’s (1910) writing informed one of the earliest and most enduring process models, developed by Wallas (1926), which describes preparation, incubation, illumination, and evaluation as four mental stages of creativity. Wallas’s (1926) model (and subsequent creativity models) often describe creativity as a combination of explicit (e.g., preparation and verification) and implicit mental processes (e.g., incubation and illumination). The physical context of creativity was largely overlooked^3^ until Rhodes (1961) proposed a 4P categorization system to organize creativity research according to its focus on *Person* (e.g., personality, attitudes, habits and behaviors), *Process* (e.g., mental processes and cognitive mechanisms, motivation, and perception), *Product* (e.g., artifact classification and evaluation) or *Press* (e.g., social and physical environment, including social climate and architectural atmosphere and people’s responses or reactions to them).

Around this time, Jonas Salk engaged the architect Louis Kahn to design the Salk Institute as a setting to enhance scientific discovery (Leslie, 2008, 2010). Salk believed that architecture could play a powerful role in fostering creativity. During a period of creative block, he had a breakthrough leading to a cure for polio while staying at an Italian monastery. Crediting its architecture as contributing to the breakthrough, he tasked Kahn to design a research facility that would similarly ignite the creativity of the scientists who would work there (Kahn, 2003). The design strategies Kahn developed are still prevalent in commercial and academic buildings today: inspirational exterior and interior settings to foster ideation; flexible, reconfigurable architecture to accommodate different creative practices; and spatial configurations that link destinations with hallway nooks, sunken gardens, and courtyards to encourage social creativity (Kahn, 2003, p. 71; Leslie, 2008). Although Rhodes’s (1961) 4Ps introduced the concept of creative Press, researchers were slow to consider it an area worth empirical investigation. Furthermore, it was designed as conceptual organizer and did not propose to offer a structure to facilitate new areas of research, including integrative approaches, despite being often referenced in calls for such efforts (Glăveanu, 2013).

Since the 1980s and corresponding with the rise of technology companies and their need for knowledge workers^4^, modern office buildings^5^ have popularized conceptualizations of how interior designs might enhance employee social creativity (e.g., through open work and touch-down areas) and attract creative talent with unexpected and playful elements (Dul et al., 2011; Thoring et al., 2020; Maier et al., 2022). Competitive advantage and economic pressures had companies embracing interior design as key component of their innovation strategy (Suckley and Nicholson, 2018). In parallel, Amabile’s (1983) componential model^6^ brought attention to the social environment in organizational creativity and was soon followed by others reconceptualizing creativity as socially *embedded* (Amabile, 1996; Csikszentmihalyi, 1996; Feldman et al., 1994) and, more recently, *distributed* across groups, creations, and locations (Glăveanu, 2014; Sawyer and DeZutter, 2009), as a form of *extended cognition*.

### Material press

Some research aligning with *embodied* and *enactive* cognition emerged in the late 20^th^ century, examining how people incorporated physical artifacts into problem solving processes (e.g., Schön, 1983; Suchman, 1987) and how creative *flow* (Csikszentmihalyi, 1996) is enacted through engagement with tools and materials. *Flow* drew attention to the role of making in fostering a “phenomenological model of consciousness” (p. 25) involving a “dynamic interplay between individual abilities and environmental opportunities” (Csikszentmihalyi et al., 2018, p. 215). Csikszentmihalyi (1996, 2018) described how people use their physical environment as both a precondition for *flow* (by creating comfortable, habitual settings) and as a resource to sustain *flow* by providing feedback on creative thinking-in-action through tools and materials or protecting the person from interruptions that inhibit the sustained focus required for flow. Although he professed the physical environment was likely important for creativity, Csikszentmihalyi (1996, p. 135) questioned whether it might be “impossible to examine empirically.”

Increasing awareness of the role of action as part of the creative process is evident in the rising popularity of makerspaces and academic incubator buildings on university campuses. The Massachusetts Institute of Technology is often credited with developing the first academic makerspace in 2001, the Fab Lab founded by Neil Gershenfeld as part of his Center for Bits and Atoms (Stacey, 2014). Although the Fab Lab was initially conceptualized as a resource for Gershenfeld’s research team, it soon became a popular setting for students to explore and solve a wide range of creative problems. Academic Incubators are a recent (and increasing popular) building type constructed on university campuses to provide a physical context, curriculum, and culture supporting multi-disciplinary creative collaboration through experimentation, hands-on skills acquisition, and strategic industry partnerships (Delphenich and Broz, 2015; Liu and Ruming, 2024). They include makerspaces to prototype and fabricate with equipment like 3d printers, laser cutters, and other tools for working with a wide range of materials – and often include areas for intentional or serendipitous collaboration, such as co-working areas, comfortable lounge-style “touch-down” areas, meeting and project rooms, and cafes. However, these physical platforms for creativity remain understudied in terms of theoretical grounding, research-informed design strategies, and post-occupancy impacts on users’ creativity – with what research that does exist dominated by qualitative case studies focusing primarily on software and fabrication technologies (e.g., 3d printing) with little exploration of spatial qualities (Soomro et al., 2022; Sharma and Halder, 2023).

More recently, Glăveanu (2013) recognized the role of the socio-material environment with his 5A’s framework, informed by ecological psychology and the work of James and Eleanor Gibson. Inspired by the 4Ps, it describes creativity as a socio-cultural phenomenon that is relational and distributed, involving Actors, Audiences, Artifacts, Actions, and Affordances as parts of the creativity system. Action is used to replace Process in Rhodes’s (1961) framework, and is defined as “both psychological and material, internal and external, goal-directed, structured, and symbolic or meaningful” (p. 73). The term Artifact is used to draw attention to the “cultured nature” of creative products. Audience replaces social Press of the 4Ps and Affordance replaces material Press. Glăveanu (2013, 2014) describes a “sociocultural presentation” of the concept of affordances (p. 76), explaining how people’s abilities to perceive and exploit affordances (which he defines as relational opportunities for action) are bound by socio-cultural norms. He argues that creativity often happens at the fringes, by norm-violating, or by translating a mundane affordance into a new context, which changes its action opportunities. Affordances are considered with respect to the tools, materials, and resources available in a creative situation, drawing attention to objects as *things to think with* and not necessarily the physical environment in which situated actions take place. Nonetheless, Glăveanu (2013, 2014) does suggest that Affordances can be “material-instrumental, functional, communicative, and symbolic” (p. 75) and so the concept might be extended to creative placemaking. However, similar to Rhodes’s (1961) 4Ps, Glăveanu’s (2013, 2014) 5A’s framework does not describe relationships between A’s and is therefore not a model that can be directly applied to environmental design strategies. Instead, he proposes that it be used to guide research, which he felt the 4P’s categorization could not do (p. 78), and, importantly, suggested that *the most basic unit of analysis should be the interaction between two or more A’s* (p. 70).

### Person-environment creativity research

The onset of the 21^st^ century saw a growing interest in designing settings for creativity, including a plethora of coffee table books about office (e.g., Borges et al., 2013; Groves and Knight, 2013; Stewart, 2004) and educational facility designs (e.g., Cannon Design, V.S. Furniture, and Bruce Mau Design, 2014; Dudek, 2012; Mirchandani, 2015). In parallel, researchers began utilizing interview, survey, or case study methods to better understand people’s perceptions about what makes a building or interior creative (e.g., Ceylan et al., 2008; Dul and Ceylan, 2011; Kristensen, 2004; Lewis and Moultrie, 2005; McCoy and Evans, 2005). More recently, there has been an uptick in experimental research examining relationships between specific environmental qualities and creative cognitive processes. This is likely due to a growing interest in understanding how physical environment conditions impact different senses, and, in turn, affect creativity (Mehta and Dahl, 2019) as well as increased client pressures and market competition among architectural firms (Criado-Perez et al., 2020; Huber, 2021). Researchers have examined atmospheric conditions (e.g., sound, color, light, smell, temperature) and spatial design strategies (e.g., ceiling heights/volume, spatial configurations, decorative elements, furnishings, views) with respect to cognitive processes associated with ideation, typically utilizing divergent thinking, convergent thinking, and insight tasks. For example, darker lighting conditions (Steidle and Werth, 2013) and moderate levels of background noise (Mehta et al., 2012; Awada et al., 2022) were found to benefit abstract conceptualization and improved overall creativity, whereas blue light was found to benefit convergent thinking (Abdullah et al., 2016) and warm-colored light to benefit divergent thinking (Weitbrecht et al., 2015). Studies examining spatial designs found less-confined areas (e.g., with higher ceilings, uncrowded with people) benefited creativity by facilitating abstract and relational thinking (Meyers-Levy and Zhu, 2007; Tong and Li, 2021) whereas more-confined areas (e.g., narrow widths between walls) induced greater variety-seeking and unique choices (Levav and Zhu, 2009). These studies suggest that spatial configurations may facilitate some creative cognitive processes while inhibiting others. Furthermore, atmosphere should be considered in terms of both quality and intensity. For example, Mehta et al. (2012) found that participants performed better on a Remote Associate Test (RAT) when background noise of coffee shop recordings was at 70 dB (versus 50 dB or 85 dB) whereas Awada et al. (2022) found white noise at 45 dB benefited RAT performance but at 65 dB it increased participants’ stress, negatively impacting creativity. A few studies have found that those settings that people identify as “creative” do seem to positively influence creativity (e.g., Dul et al., 2011; Dul and Ceylan, 2011; McCoy and Evans, 2002), but it remains unclear why that is. There have been several recent attempts to organize the empirical literature to address this question. Instead, these have revealed diverse methodological approaches and theoretical grounding, lack of replication, and variety in how *creativity* and *creative space*^7^ are conceptualized (Blomberg and Kallio, 2022). Thus, it is difficult to draw conclusions about the role of the physical environment in creativity.

### Summation: awareness is a circular problem

**Figure 1:**
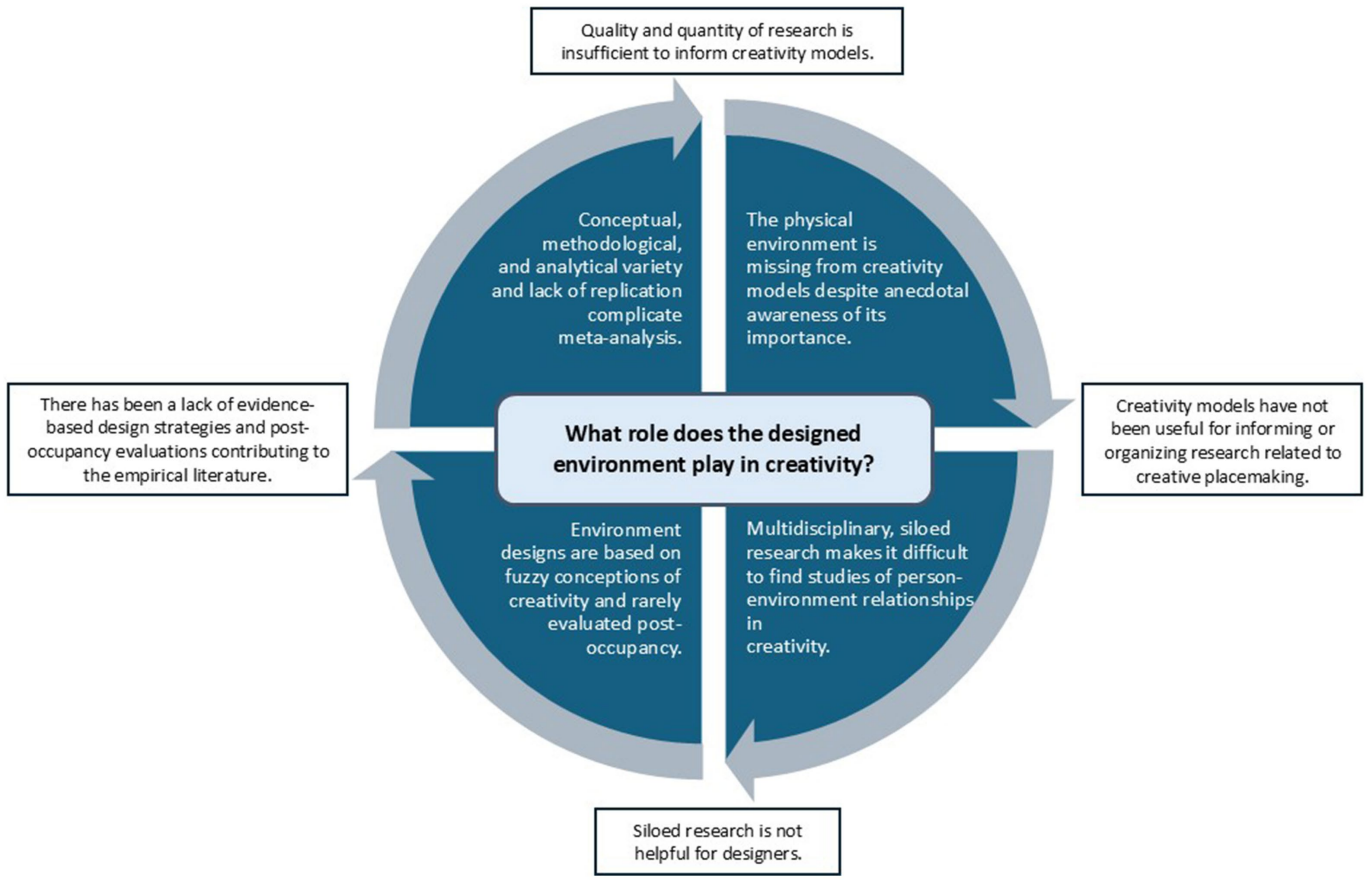
The awareness problem is circular.

To summarize (Figure 1), there is significant anecdotal and some qualitative evidence that creative people identify certain settings as “creative” and that they feel materials, resources, atmospheres, and spatial configurations play a role in creative processes and productivity. Yet, existing models of creativity do not attempt to describe person-environment relationships during creative endeavors, mainly focusing instead on creativity as mental stages or socially-situated processes. Still, environmental designers and organizations consider the designed environment a strategic resource to enhance individual and social creativity. However, placemaking efforts are often based on unclear conceptualizations of creativity and post-occupancy empirical examination of design impacts are rare. This is likely because (a) the physical environment is largely missing from creativity models, (b) existing research is published in a wide range of academic journals, making it difficult to find, and (c) methodological and analytical approaches are diverse and lack replication of findings. Furthermore, there is significant variety in how “creativity” is conceptualized through environmental design, making it difficult to draw conclusions about design practices or understanding how the physical environment might fit within current creativity models.

## The definition problem: creativity as physically situated practice

Creativity and innovation are closely related concepts and are often used interchangeably (Tang, 2017), particularly in organizational, educational, and professional contexts. Creativity research has mostly focused on how people come up with novel and useful ideas whereas innovation is often defined as “successful implementation of creative ideas by organizations” (Amabile, 1988, p. 126; Forgeard and Kaufman, 2016, p. 2). However, it is now generally accepted that creativity is involved in everything from problem finding through implementation – blurring distinctions between creativity and innovation (Caroff et al., 2018). Although there is strong consensus around the “standard” definition of creativity, it primarily describes creative products (e.g., as novel and useful). Creative “ability” is referenced, but there is a lack of specificity in how creativity is enabled, constituted, operationalized, or developed. Thus, a need for greater specificity in defining and operationalizing this complex psychological construct is widely recognized within the field (e.g., Kenett et al., 2020; Kaufman and Glăveanu, 2021; Green et al., 2023). Furthermore, for a definition to be of use to environmental designers, it should ideally describe embodied and situated aspects, so that designers are not left to bridge the gap between mental processes and physical environments.

### Creativity as mental processes

**Figure 2:**
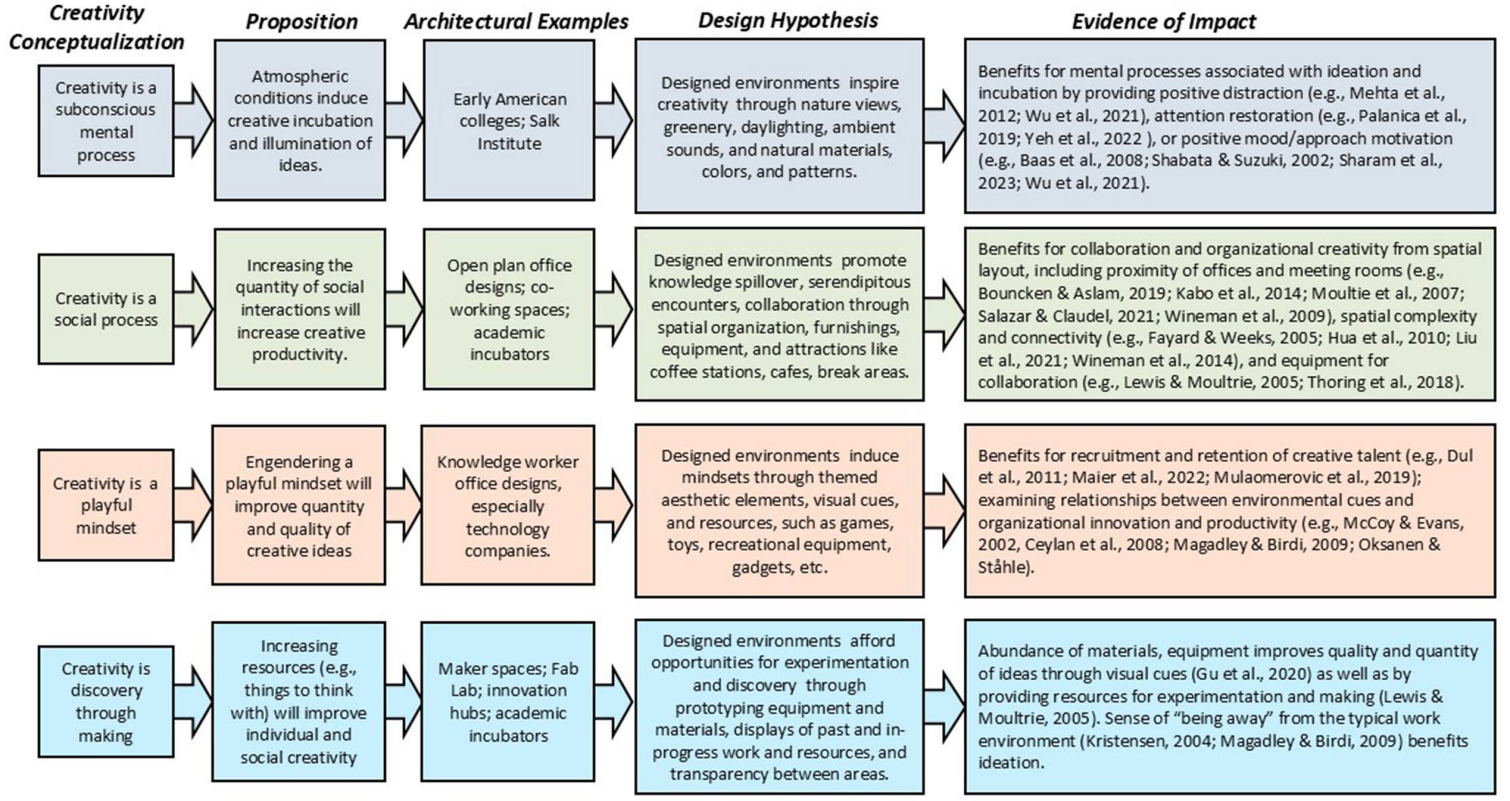
How creativity is conceptualized through environmental designs.

Indeed, when it comes to creative placemaking, designers’ conceptualizations of creativity vary (Figure 2), with strategies predicated on creativity as primarily (a) an individual or social activity, (b) a mindset or a series of mental stages, or (c) involving subconscious or conscious work on a problem – which, in turn, informs assumptions about whether designed environments directly or indirectly affect creativity. Architectural strategies emphasizing views of nature and inspirational aesthetics and atmospheres conceptualize creativity as unconscious (or subconscious) work, suggesting environmental conditions can help induce incubation and benefit illumination of creative ideas. The largest body of research corresponds with this conceptualization, including experimental studies examining impacts on ideation from views and access to nature (e.g., Palanica et al., 2019; Sharam et al., 2023; Yeh et al., 2022), indoor plants (e.g., Chulvi et al., 2020; Shibata and Suzuki, 2002; Studente et al., 2016), lighting (e.g., Abdullah et al., 2016; Lan et al., 2021; Steidle and Werth, 2013), interior colors, patterns, and materials (e.g., Stone, 2003; Studente et al., 2016, Vohs et al., 2013) room shapes (e.g., Meyers-Levy and Zhu, 2007; Strachan-Regan and Baumann, 2024; Wu et al., 2021) and ambient sounds, temperature, and smells (e.g., Awada et al., 2022; Ijzerman et al., 2014; Knasko, 1992; Mehta et al., 2012). Although there is little research examining whether environmental atmospheres can engender specific creative states, some studies hypothesize that designed environments mediate mental processes by providing positive distraction/reduced mental focus (e.g., Mehta et al., 2012; Wu et al., 2021), restoration of directed attention (e.g., Palanica et al., 2019; Yeh et al., 2022), or positive mood/approach motivation (e.g., Baas et al., 2008; Shibata and Suzuki, 2002; Sharam et al., 2023; Wu et al., 2021) benefitting incubation and ideation.

### Creativity as social processes

Creativity is conceptualized as a socially-situated process through designs that encourage interpersonal interactions, emphasizing open work areas, movable furnishings, and settings that attract people through food, drink, or break areas. This strategy is associated with research utilizing survey, interview and observational methods examining impacts on collaboration and organizational creativity from interior layout, including proximity of offices and meeting rooms (e.g., Bouncken and Aslam, 2019; Kabo et al., 2014; Moultrie et al., 2007; Salazar Miranda and Claudel, 2021; Wineman et al., 2014), spatial complexity and connectivity (e.g., Fayard and Weeks, 2007; Hua et al., 2011; Liu et al., 2021; Wineman et al., 2014), furniture design and postures (e.g., Knight and Baer, 2014; Setola and Leurs, 2014; Malinin, 2019), and equipment for collaboration (e.g., Lewis and Moultrie, 2005; Thoring et al., 2018).

### Creativity as playful mindset

Coffee table books often depict designs where creativity is conceptualized as “play” (e.g., Borges et al., 2013; Cannon Design, V.S. Furniture, and Bruce Mau Design, 2014; Groves and Knight, 2013; Stewart, 2004), illustrating “symbolic” design strategies that incorporate themed interiors with unexpected elements, environmental cues, and areas for recreation and leisure – particularly those emphasizing childhood associations, such as over-scaled staircases, primary or bright colors, indoor slides, etc. These designs sometimes encourage extreme personalization, suggesting the organization values individualization and freedom from convention (Amabile et al., 2002; Elsbach and Pratt, 2007). The corresponding small body of research related to this approach is generally concerned with how designs affect creative talent recruitment and retention (e.g., Dul et al., 2011; Maier et al., 2022; Mulaomerovic et al., 2019) or how environmental cues affect organizational innovation and productivity (e.g., McCoy and Evans, 2002; Ceylan et al., 2008; Magadley and Birdi, 2009; Oksanen and Ståhle, 2013). Although design strategies related to play typically fall under “symbolic” approaches, they sometimes encourage movement and unconventional postures (e.g., meeting areas with bean bag chairs) to shape creative thinking.

### Creativity as discovery-through-making

Finally, “making” is promoted through designs conceptualizing creativity as a process of discovery arising through interactions with materials. Corresponding research typically involves case studies of corporate innovation labs (e.g., Fecher et al., 2020; Kristensen, 2004; Magadley and Birdi, 2009) or academic makerspaces (e.g., Fleischmann et al., 2016; Forest et al., 2014; Lam et al., 2021). There is some overlap between conceptualizing creativity as “fun” and “making” because making is often considered fun (Lee, 2016) and areas for making can provide a sense of “being away” from the typical work setting (Kristensen, 2004; Magadley and Birdi, 2009). Furthermore, areas for making can be perceived as visually complex, or “junk laden” (e.g., abundance of material and equipment), which can improve the quality and quantity of ideas through visual cues (Gu et al., 2020) as well as provide resources for experimentation (Lewis and Moultrie, 2005).

### Summation: creativity as embodied-enactive experience

There remain unanswered questions about the role the designed environment plays in creativity, including whether it (a) indirectly affects creativity by engendering physiological responses influencing mood, mindset, or cognitive processes, (b) shapes behaviors by visually communicating expectations for collaboration or making, (c) provides resources that extend creative cognitive abilities, − or if it is perhaps co-constitutive of a person’s cognitive system. Recently, some theoreticians have turned to embodied cognitive science to provide theoretical grounding to better understand person-environment relationships. *Embodied-enactive architectural experience* (Jelić et al., 2016; Rietveld and Rietveld, 2018) proposes that designed environments enact embodied cognitive processes through relational coupling between person and environment. An interdisciplinary emerging stream of *embodied creativity* research, grounded in ecological psychology and cognitive neuroscience, aims to better understand the body’s role in creative cognitive processes by examining bodily postures and interactions (Frith et al., 2020; Kimmel and Groth, 2024; Malinin, 2019). Experimental studies have demonstrated that free walking (Kuo and Yeh, 2016; Leung et al., 2012), open body postures (Andolfi et al., 2017; Hao et al., 2014), fluid arm movements (Slepian and Ambady, 2012), and working in a warm setting (Ijzerman et al., 2014) benefit divergent thinking whereas moving the arms together (Leung et al., 2012), or squeezing a hard ball (Kim, 2015) were found to improve convergent thinking. Additionally, holding warm objects was found to improve relational thinking and higher quality, realistic ideas whereas holding cool objects improved metaphorical, divergent, and abstract ideation (Ijzerman et al., 2014). Studies such as these raise new questions about ways creativity might best be empirically examined. The embodied-enactive architectural perspective may provide a path forward towards redefining creativity by extending Glăveanu’s (2013, 2014) concepts of Action and Affordance to environmental design, bringing attention to the possible relationships between them. For example, interior design might shape mental processes, encourage or discourage social interactions, influence mindsets, or enact discovery and exploration through making. Similarly, this coupling of Action and Affordance may prove useful for conceptualizing creative *space* and *place*.

## The conceptualization problem: creative spaces and places

How people perceive *space* and *place* is a point of philosophical debate among theorists. However, for architectural designers it is a practical concern essential to understanding user experiences – and for their clients it is an economic issue. The designed environment is increasingly considered by real estate developers, facility planners, and corporations as a *strategic tool* to enhance user creative productivity, driving organizational innovation and growth (De Paoli et al., 2013). The concept of creative placemaking inherently brings together different disciplinary perspectives: spatial design, person-environment theories, and creativity research. Thus, to address the *awareness* and *definition* problems highlighted in the prior section of this paper, it is also important to consider the various ways designers and researchers conceptualize *space*, *place*, and *environment*.

### Behavior settings

From the environmental psychology perspective, creative working and learning areas might be considered *behavior settings*. Barker (1968), one of the founders of the field of ecological psychology, developed the theory with Wright, based on observations that human behavior is best predicted by the setting in which it occurs. In other words, groups of people behave more similarly in a particular place (e.g., church, school, work, etc.) than if you compared their behavior across those different settings. Similar conceptually, Duffy et al. (1998) proposed four typologies to describe event-space patterns of office worker behavior. The “cell” is a private office (or highly screened workstation) designed for concentrated, focused working. The “hive” describes clustered workstations for individual process work, organized by team or type of work. The “den” is a meeting area for groupwork. Finally, the “club” describes flexible areas designed to support a variety of tasks associated with transactional knowledge working. Activity-settings are often used in modern offices, providing a variety of different designs for focused work (e.g., quiet nooks, sound-proofed pods, self-contained closed-door rooms, and phone booths) and collaborative activities (e.g., lounges, cafes, open work areas, conference rooms, huddle rooms, pitch rooms and project rooms). More recently, activity-based flexible office (AFO) designs have been popularized to attract and empower knowledge workers – although a primary motivation for organizations has been to reduce facility costs (Parker, 2016). Workers have no assigned work area in AFOs; they are expected to change locations based on work activity. The lack of dedicated work area yields planning efficiencies, because they account for percentage of workers to be away on leave or work-related travel, thus these designs use less square footage and fewer furnishings than conventional office designs. A recent review of 23 activity-based office studies found that, when compared to office designs with assigned work areas (cellular, mixed-assignment, and small or medium-sized open-plan layouts), activity-based offices were generally less favorable on all dimensions, including environmental satisfaction, social relations, cognitive performance, productivity, and job satisfaction (Masoudinejad and Veitch, 2023). However, the small number of studies and high variety in office designs and workers make it difficult to draw any conclusions about whether AFOs might benefit creative work. Furthermore, these studies do not account for personal spatial constructs, such as territoriality and place attachment (Frankó et al., 2023).

The ecological psychologist Heft (2024) has argued that, although behavior setting theory has fallen out of favor among psychologists, it may have renewed relevance with respect to recent developments in 4E cognitive science. Behavior settings describes dynamic and nested emergent eco-psychological systems operating in human habitats, which aligns with modern ecological approaches to cognition. Heft (2024) suggests that behavior settings function similarly to *affordances*, as attractors for engagement, thereby fostering “functional interdependencies among those individuals that sustain their actions with the support of material features (e.g., affordances) present” (p. 5). Finally, he recommends that behavior setting theory would benefit from greater consideration of both the role of the individual – including improvisational, situated actions – and the features and qualities of a setting that support patterns of activities, toward better understanding their roles in maintaining and reshaping behavior settings. Thus, Heft (2024) seems to be suggesting some level of integration between behavior setting theory and affordance theory, which are often considered distinct. And, while behavior setting theory has “been all but forgotten” (p. 1) among modern psychologists, affordance theory has seen a resurgence in popularity in cognitive science, creativity research, and architectural design.

### Affordance, umwelt, habitat

Gibson’s (1977) affordance theory describes how visual perception is constituted through person-environment relationships, distinguishing between an individual’s environment (*umwelt*) and a group’s environment (*habitat*). He borrowed the concept of umwelt from von Uexküll (2010), who explained that an organism exists at the center of its umwelt, which is defined by that organism’s abilities and senses. Thus, the organism is conceptually bound to its umwelt and, even if several organisms exist together in a habitat, each has its own umwelt. von Uexküll (2010) introduced the concept of *funktionale Tönung* (functional coloring) to describe the latent action possibilities of an object in an environment as related to the ability of an organism to exploit them. Gibson (1976, 1977) extended these concepts to describe *affordance*, an opportunity for action that exists through person-environment coupling. For example, a table on wheels affords moving. A person may *perceive* this affordance, and not *actualize* it (i.e., move the table). If a person does not perceive the affordance, it still exists but is *hidden* to them. Although Gibson (1976) was especially interested in how his affordance theory could be used to understand how architectural designs might shape or constrain behaviors, Baggs and Chemero (2019, pp. 5–6) point out that he was ambiguous in the way he referred to the environment – as the physical world, as habitat, or umwelt. And this ambiguity, in turn, has led to confusion about whether an affordance is a relationship between individual and umwelt or a property of the habitat for a group of people.

The concept of affordances has been applied to a variety of design fields, including product design, interaction design, social media, and architecture, to guide design strategies and usability research. Norman (2008) was interested in how designed objects and environments might communicate their intended use; thus he was concerned primarily with perceivable affordances, and he later coined the term *signifier* to refer to an aspect of a designed artifact that cues (or nudges) desired behavior (p. 18). Others have proposed categorizing affordances according to types of interactions, such as social affordances (Heft, 2020), communicative affordances (Schrock, 2015), and technological affordances (Gaver, 1991). Philosopher Eric Rietveld has explored affordance theory with respect to architecture though his practice with architect Ronald Rietveld (Rietveld and Rietveld, 2018; Staničić and Jelić, 2022). Rietveld and Kiverstein (2014) proposed distinguishing between a *field of affordances* to describe those that are relative to an individual and the *landscape of affordances* as those available to a group defined by “relatively stable and regular patterns of activity found among individuals taking part in a practice or a custom” (Kiverstein et al., 2021, p. 2289). Finally, they emphasize that while there are conceptual benefits to considering individual and group environments as distinct, ultimately, they are interdependent.

### Lived, practiced, and designed space

Considering person-environment relationships with respect to individuals and groups may be particularly useful for informing creative placemaking in architectural design; however, none of the forementioned conceptualizations distinguish between natural and designed environments. Grounded in a phenomenological perspective, philosopher and sociologist Henri Lefebvre (1991) proposed a “conceptual triad” to explain how space is “produced” through embodiment (“the lived”), subjectivity (“the practiced”), and design (“the conceived”). *Lived space* describes how space is enacted through a person’s interactions in a setting, shaping the meaning and values attached to that space. *Practiced space* describes how space is co-constituted through routine social activities in a setting. *Conceived space* describes how space is explicitly designed, structured, and intended to be used, as conveyed through design elements (e.g., furnishings, resources, and environmental signs and symbols) as well as organizational policies and practices governing space usage. Lefebvre’s (1991) conceptual triad provides a structure that may be helpful for understanding the place of behavior setting theory in environmental design and research (i.e., as related to practiced space), by bringing attention to subjective experiences of lived space, including affective experiences related to place-making (Pierce and Martin, 2015). Although Lefebvre’s (1991) spatial triad describes the intertwined, dynamic interactions between perceived, lived, and conceived space, it does not address the “nested dynamics” that Heft (2024) elucidates as a strength of behavior setting theory. Additionally, it is focused on the social production of space, lacking the action-oriented focus of modern cognitive science and Gibson’s (1976, 1977) affordance theory.

### Summation: reconsidering creative press

**Figure 3:**
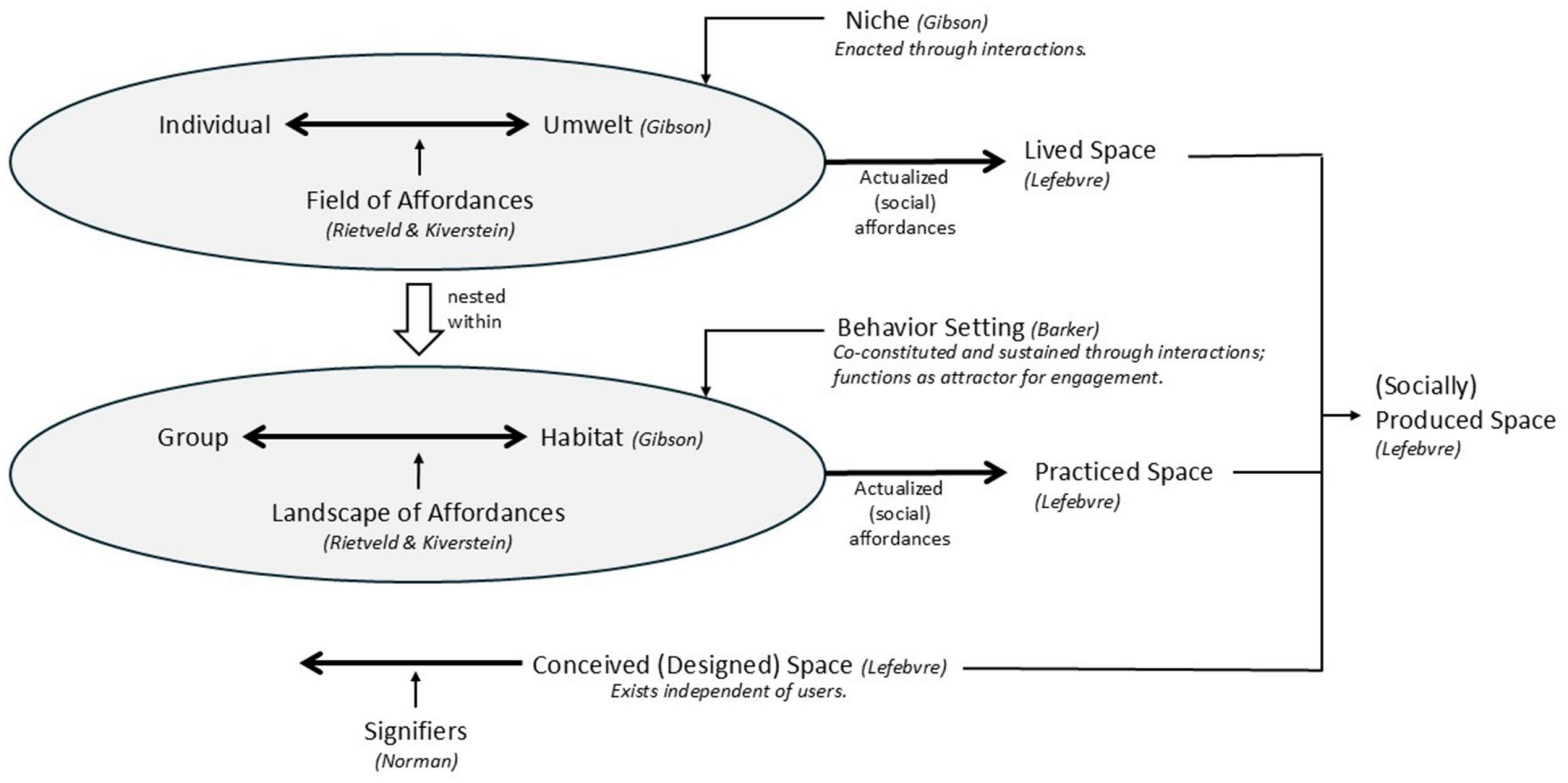
Integration of key concepts from Gibson (1976, 1977), Barker (1968), Lefebvre (1991), Norman (2008), Rietveld and Kiverstein (2014).

Creative Press, as defined by Rhodes’s (1961), is a broad categorization to describe the social and physical environments of creativity, which, Glăveanu (2013, 2014) points out, has not been useful for guiding empirical examination. Although Glăveanu (2013, 2014) proposes the 5A’s as an improved structure for guiding creativity research, its socio-cultural perspective leaves Physical Press conceptualizations underdeveloped. Environmental (e.g., behavior setting theory), ecological (e.g., affordance theory), and sociological (e.g., spatial triad) perspectives underscore the importance of considering the intertwined aspects of people and environments, suggesting that considering the Physical Press as fixed, immobile, and existing separately from users may not be productive for advancing research in this area. In summary, as a starting point for better understanding the role of the physical environment in creative processes and to guide creative placemaking in architectural designs, behavior setting theory, affordance theory, and Lefebvre’s (1991) spatial triad framework each offer key concepts (Figure 3) that might prove helpful. These include: (a) the conceptual distinction between individual/lived, group/practiced and conceived/designed environments; (b) an embodied action-oriented perspective grounded in affordance theory (and 4E cognition) tied to these conceptual distinctions (e.g., field of affordances, landscape of affordances, and signifiers of designed artifacts); and (c) a nested, dynamical systems approach. Finally, relationships should be considered between creative placemaking and associated constructs (e.g., Bernardo et al., 2023), such as place identity with respect to creative productivity and the role place attachment with respect to creative process and creative identity – given the intertwined nature of these concepts.

## Discussion: toward embodied-dynamics of creative placemaking

**Figure 4:**
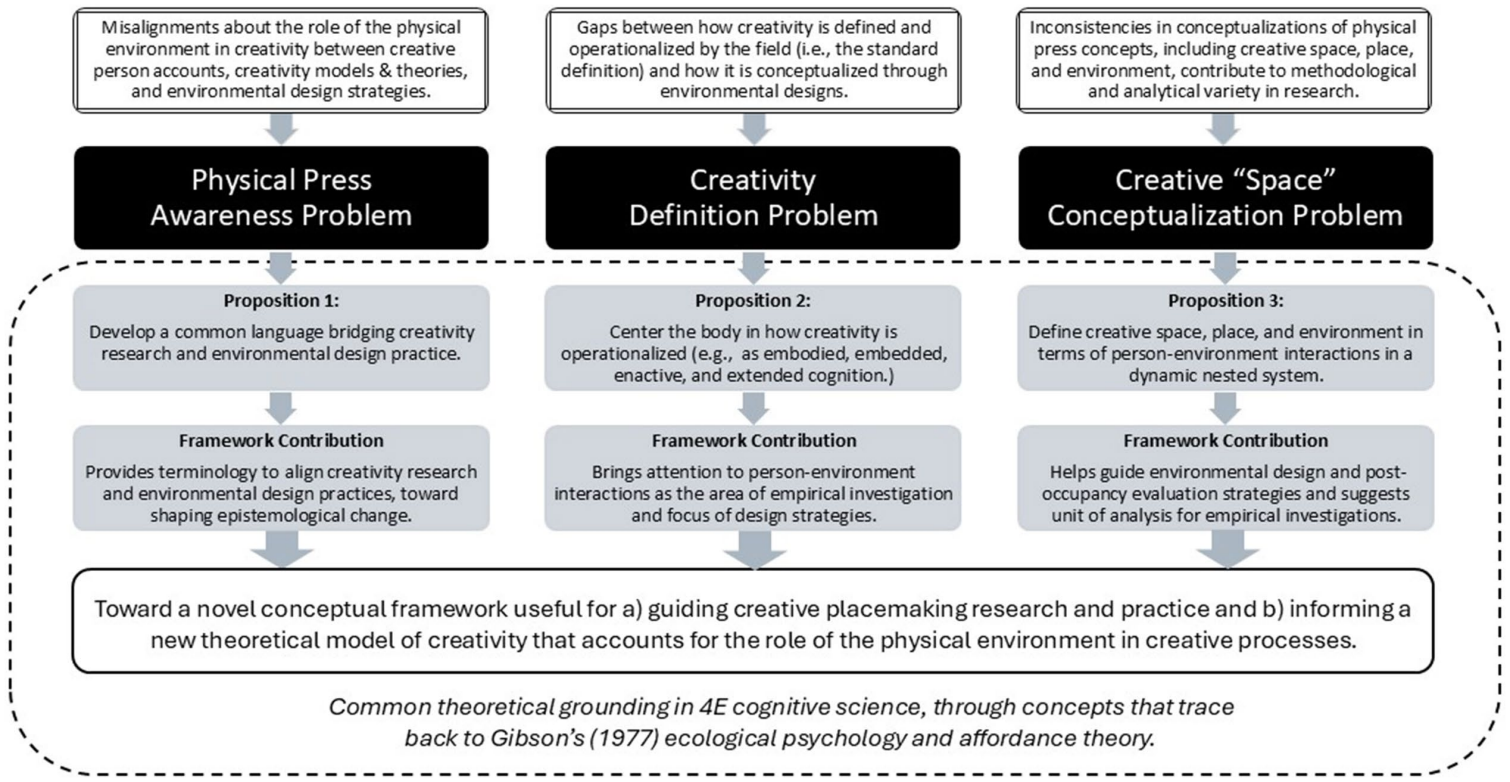
Toward understanding the role of the designed environment in creativity – problems, propositions, and contributions to inform a novel framework.

To help remedy the awareness, definition, and conceptualization problems, following are three propositions to lay the groundwork for a novel framework useful for guiding creative placemaking research and design (Figure 4). Grounded in 4E cognitive science, with concepts tracing to Gibson’s (1976, 1977) affordance theory, these propositions suggest a starting point to better clarify person-environment relationships during creative endeavors. Together they are meant to provide a structure for an integrative framework, bridging interdisciplinary research from creativity, environmental design, ecological psychology, and cognitive science with environmental design strategies intended to foster user creativity.

### Proposition 1: address the “awareness” problem through new vocabulary

**Figure 5:**
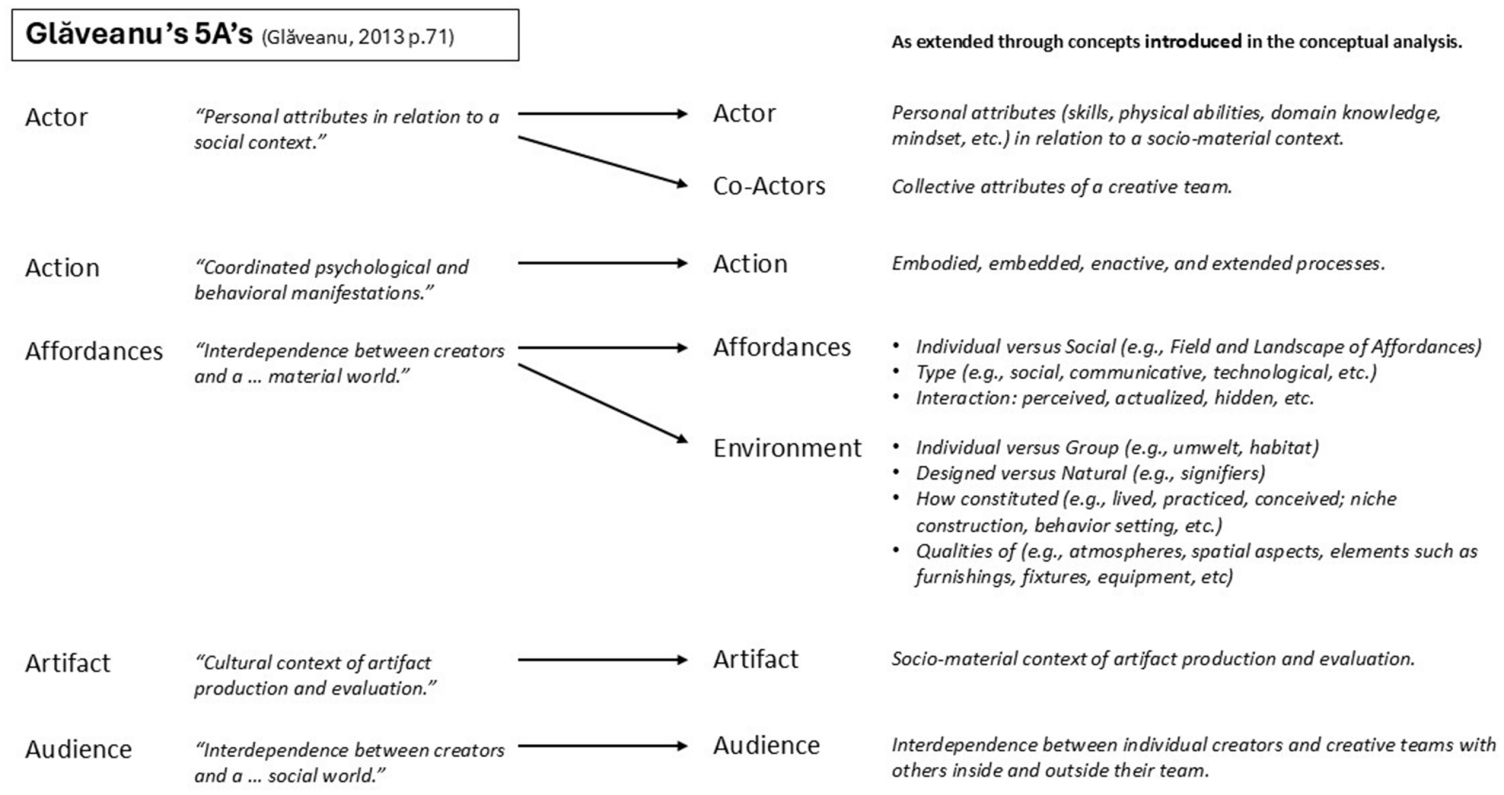
Example of how a new vocabulary bridging creativity and environmental design might be developed.

With his 5A’s framework Glăveanu (2013) introduced a new vocabulary to the creativity field that utilizes the concept of affordance to emphasize “the interdependence between creators and a social and material world” (p. 71). He suggests the 5A’s are merely a starting point, with new terminology needed to continue to shape a change in “epistemological position,” because “vocabularies [are] highly consequential for how we define, discover, assess, validate, and practice creativity (Glăveanu et al., 2023, p. 7). This first proposition similarly suggests that a new vocabulary, bridging the languages of creativity and environmental design. For example, Figure 5 uses the 5A’s as a starting point to illustrate how a common vocabulary might develop. Common terminology would make it easier to find research that has historically been published in peer-reviewed journals from a wide range of different disciplines. This, in turn, would help environmental designers collect, assess, and apply research findings through their architectural and interior designs. Aligning research and design efforts toward common goals might then begin to address the meta-analysis problem, and, consequently, provide sufficient evidence to inform creativity models, and so on.

The recent interest in affordance theory among creativity researchers provides a unique opportunity to fulfill Gibson’s (1976, 1977) aspirations that it provide insights to inform architectural design. Glăveanu (2013) proposes that creativity arises from person-environment interactions, with people first perceiving and actualizing more obvious affordances in their environments, eventually learning to master the ability to perceive less obvious affordances (through learning), and, finally, altering, adapting, and creating new affordances (p. 76). This conceptualization might reframe how designers consider creative placemaking and the role of learning in environmental designs. Norman’s (2008) affordance conceptualization, more familiar to designers, brings forth the idea of *signifiers*, underscoring the importance of design in how people perceive and actualize affordances during creativity. Finally, Rietveld’s distinction between the field and landscape of affordances would extend Glăveanu’s (2013) conceptualization to consider affordances from the individual and group perspectives, providing opportunities to explore how some affordances might, for example, constrain individual creativity while benefitting social creativity (or vice versa). Although there are a variety of sometimes-conflicting ways that affordances are conceptualized in the literature, provided here are a few suggestions for integrating complementary perspectives to guide a common language and organize efforts to better understand person-environment relationships during individual and social creativity. With its emphasis on action and affordances, the 5A’s framework is one starting point from which to imagine a more expanded taxonomy to include related concepts from environmental design and creative placemaking concerns, such as sensitivity to the body (e.g., ergonomics, anthropometrics, postures, movement, etc.), aesthetics, materiality, spatial and atmospheric qualities, and so forth.

### Proposition 2: address the “definition” problem by centering the body

**Figure 6:**
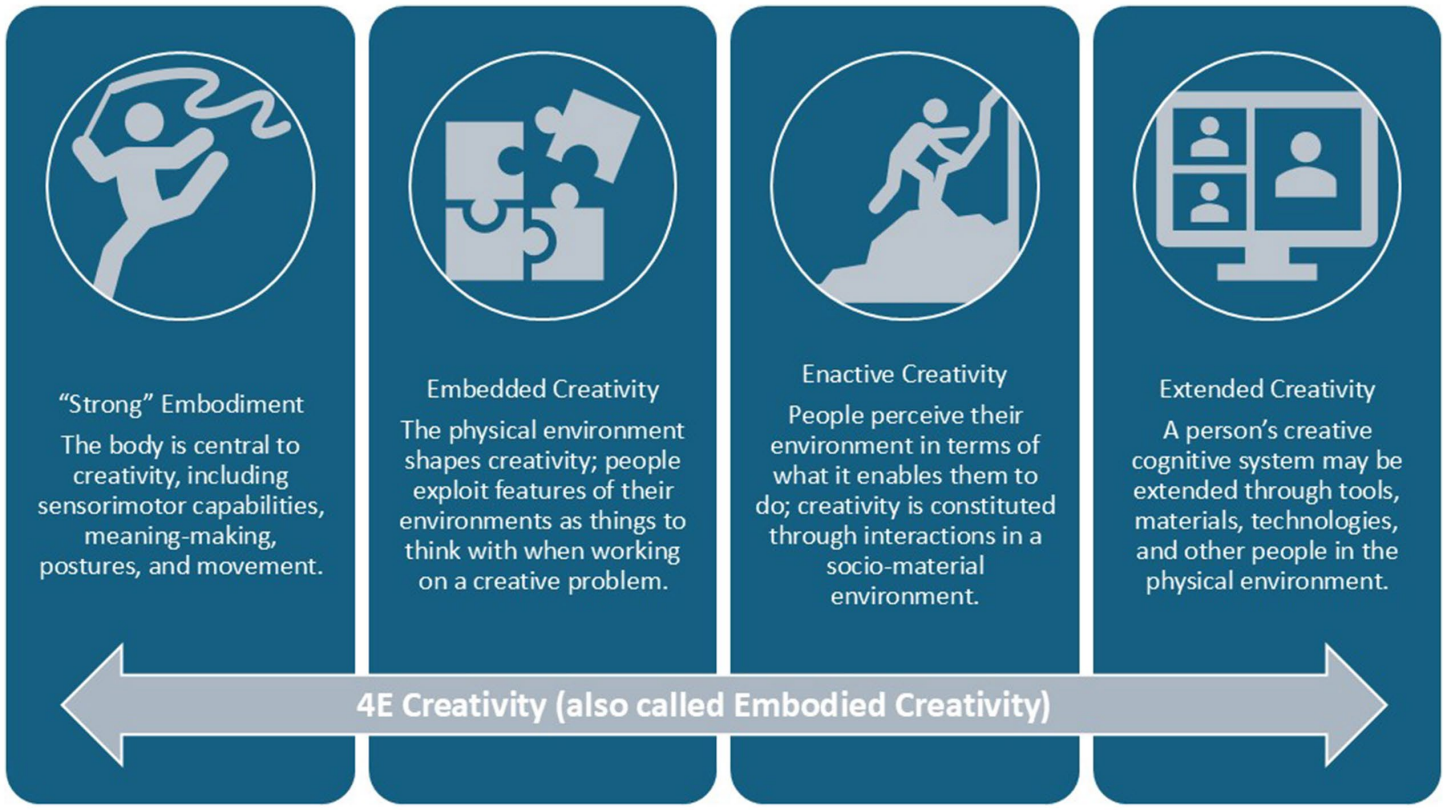
Creativity as embodied, embedded, enactive, and extended cognition.

Considering creativity and creative placemaking with respect to affordance theory brings further awareness to the misalignment between the standard definition of creativity and how creativity is operationalized as a dynamic process constituted through body-environment interactions. Therefore, placing the body at the center of situated action may provide a starting point for defining and operationalizing creativity in a way that is useful for informing creative placemaking research and practice. 4E cognitive science, as a recognized theoretical framework with concepts grounded in affordance theory and ecological psychology, is used here (Figure 6) as a lens to begin to “flip the script” on how creativity is operationalized in terms of bodily actions. For example, the previously mentioned psychological experiments demonstrating how postures, movements, and environmental atmospheres affect divergent and convergent thinking and insight tasks lends empirical support that creativity is embodied. Csikszentmihalyi’s (1996, 2018) flow theory and Glăveanu’s (2013) 5A’s framework align with the idea that a creator is embedded within a socio-material environment and that creativity depends on – and is constituted through – interactions in those environments. Finally, socio-cultural models of creativity suggest how creativity is extended through the socio-physical environment; it is distributed across people, objects, and digital technologies. This proposition posits that a 4E creativity approach opens new opportunities to examine how the body explores and exploits affordances during creative efforts as well as how it reshapes the socio-material environment to surface and forge new affordances. Table 1 illustrates how 4E creativity might bridge existing research and design strategies mentioned previously (Figure 2) as well as inform future areas of research.

**Table 1:** 4E creativity and implications for creative placemaking design and research.

4E creativity	“Strong” embodiment	Embedded creativity	Enactive creativity	Extended creativity
	The body is central to creativity, including sensorimotor capabilities, meaning-making, postures, and movement.	The physical environment shapes creativity; people exploit features of their environments as *things to think with* when working on a creative problem.	People perceive their environment in terms of what it enables them to do; creative spaces are co-constituted when people think-in-action during creativity.	A person’s creative cognitive system is extended through tools, materials, technologies, and other people in the physical environment.
Key findings previously cited.	Open postures, fluid body movements, and warm temperatures and lighting (low intensity) improve divergent thinking (Andolfi et al., 2017; Hao et al., 2014; Ijzerman et al., 2014; Kuo and Yeh, 2016; Leung et al., 2012; Slepian and Ambady, 2012; Steidle and Werth, 2013). Constrictive body movements and cool lighting benefit convergent thinking (Abdullah et al., 2016; Leung et al., 2012; Kim, 2015).	Resource-rich environments promote experimentation (Lewis and Moultrie, 2005) and improve quality and quantity of ideas (Gu et al., 2020).	Creative people alter their settings or move to different settings in order to enable different creative processes (Buttimer, 1983; Fig, 2009; Malinin, 2016). Creative flow (Csikszentmihalyi, 1996) is enacted and sustained by thinking-in-action with tools and materials.	Creativity is distributed across groups of people, artifacts, and settings (Glăveanu, 2014; Sawyer and DeZutter, 2009).
Implications for creative placemaking.	Environmental designs provide sensorimotor experiences that shape embodied ideational processes (e.g., affecting divergent, abstract, relational, and convergent thinking).	Environmental designs are resources for creativity (e.g., *things to think with*).	Environmental designs cue action opportunities (i.e., through designed affordance *signifiers*), influencing how people perceive and actualize affordances during creative endeavors.	Environmental designs extend and distribute individual and group creativity by functioning as creative artifacts and through infrastructure provided (equipment, design elements and materials, technologies, etc.)
Examples of related design strategies.	D.1a. Specify artificial lighting with controls for changing color temperature and intensity.	D.2a. Plan areas for making with a variety of tools, technologies, and materials.	D.3a. Install environmental graphics in activity-focused office (AFO) designs to signify unique affordances of different areas with respect to 4E creative processes.	D.4a. Provide exhibition areas or other places to display in-process and completed creative projects.
	D.1b. Promote free walking in collaborative ideation areas through furniture selections (e.g., scattered standing-height tables) and equipment layout.	D.2b. Provide open shelving or other visible storage for inspirational objects (e.g., gadgets, construction toys, etc.) in collaborative ideation areas.	D.3b. Incorporate themed interior designs to signify organizational values and goals (e.g., 1950’s themed décor for a company that designs products for the baby boomer generation).	D.4b. Develop spatial configurations to connect work areas with “attractor” areas (e.g., break areas, recreation areas, etc.) to promote serendipitous interactions between people and artifacts.
Examples of testable hypotheses.	H.1a. Ideas generated in an environment with warm-colored light at lower intensity (dimmer) will be more original than ideas generated in an environment with cool-colored light and higher intensity light. *Experimental study.*	H.2a. Knowledge workers that have ready access to makerspaces during their workday will be more creatively productive (e.g., greater fluency, originality, elaboration of ideas) than knowledge workers without access to makerspaces. *Quasi-experimental study.*	H.3a. People working in AFOs with environmental graphics used to signify affordances will have higher levels of environmental satisfaction than people in AFOs without environmental graphics. *Quasi-experimental study.*	H.4a. Organizational knowledge and creativity is more widely distributed across workers in an office where in-process and completed projects are displayed than in a similar office where work is not displayed. *Quasi-experimental study.*
	H.2b. People who free-walk during ideation activities will produce more original ideas than people who sit. *Experimental study.*	H.2b. People will produce ideas with greater fluency and originality in environments where they can interact with inspirational objects than ideas produced in environments without inspirational objects. *Experimental study.*	H.3b. People will develop higher quality, elaborate, and realistic ideas (i.e., relational thinking) in a themed environment versus an unthemed environment. *Experimental study.*	H.4b. Offices with designed attractor areas will have a higher number of collaborative creative interactions than offices without designed attractor areas. *Quasi-experimental study.*

Operationalizing creativity with respect to 4E cognition reveals that definitions that, for example, describe creative Person and Press as separate elements of creativity, are likely unproductive for advancing the field and informing creative placemaking efforts. Creativity happens through interactions between elements, constituting a dynamic and evolving system where each element impacts the others. Although 4E cognitive science is generally considered an established research field, it is still early in development. Researchers continue to grapple with questions such as whether cognition is strongly or weakly embodied by bodily and extrabodily processes and if cognition is constituted by action or only partially dependent on action (Newen et al., 2018, p. 6). Thus, it may be somewhat premature to propose a new creativity definition grounded in 4E cognition. Nonetheless, operationalizing creativity as embodied, embedded, enactive, or extending can help organize and focus interdisciplinary efforts *towards* a definition that is useful for researchers and environmental designers.

### Proposition 3: address the “conceptualization” problem through a nested systems approach

**Figure 7:**
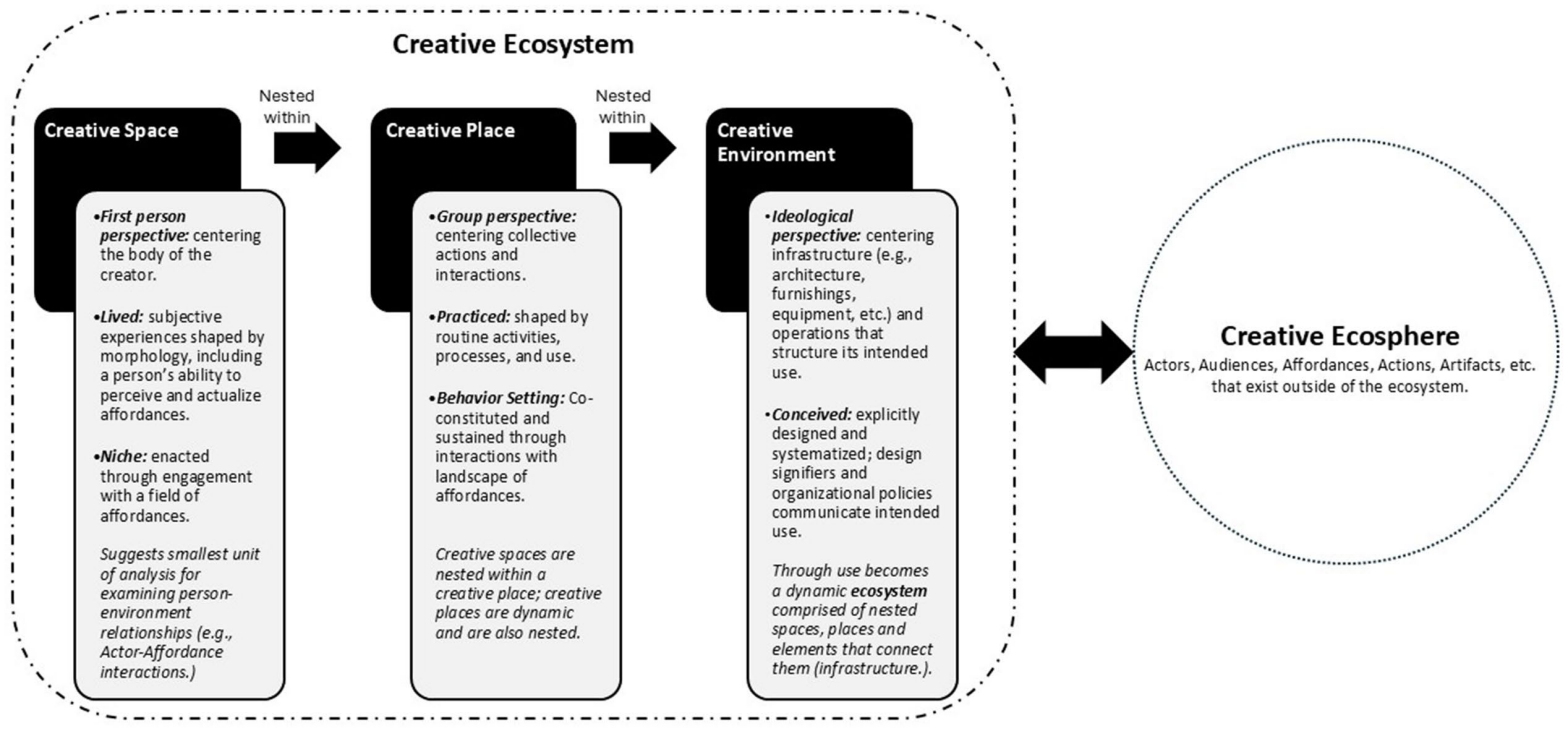
Conceptualizing the spaces, places, and environments of creativity: an embodied-enactive perspective.

If creativity is best examined in terms of interactions between people and their socio-physical environment, as a 4E approach suggests, then it becomes essential that more attention is given to how “environment” is conceptualized. Inspired by work of Barker (1968), Gibson (1976, 1977), and Lefebvre (1991) (including extended theoretical work by Heft (2024), Norman 2008, and Rietveld and Kiverstein, 2014) creativity research and placemaking design may benefit from conceptual distinctions between the individual/lived (creative space), group/practiced (creative place) and conceived/designed (creative environment) experiences as a dynamic and nested system of the architectural setting (Figure 7). Such conceptual distinctions might help shape decisions about the appropriate unit of analysis to consider when exploring questions about person-environment relationships through research studies or when developing propositions to guide design hypotheses and strategies.

*Creative Space* is related to the concepts of *lived space* and *umwelt* in that it describes the transactional relationship between an individual and their immediate socio-material environment. It takes an egocentric (first person) perspective, considering the body as central to enacting creative space. It borrows the concept of niche construction from ecological psychology, drawing attention to how people act within and on their environment in service to creative endeavors. Environment, in this conceptualization, includes architectural elements (furnishings, equipment, etc.) and atmospheric conditions (sounds, temperature, lighting, color, aroma, nature views, etc.) as well as the tools and materials of the creative endeavor. For example, a knowledge worker in an activity-based office might have a variety of workspaces – a private office, a workstation, a lounge area, a workbench, etc. Understanding how and why people choose to work in different areas with respect to creative processes, goals, affective states, abilities, etc. would help to understand how they perceive and actualize the *field of affordances* in their environment, including how their perception of environmental affordances might be enriched by skills development and situated learning. Furthermore, this conceptualization’s concern with the *lived experience* of the creator also considers meaning and personal values, including how space is subjectively imagined and experienced, as well as related concepts such as place attachment, territoriality, and place identity.

*Creative Place* conceptually integrates concepts of *practiced space* and *habitat,* with behavior setting theory, extended through Rietveld’s *landscape of affordances* as potential action opportunities in a shared or public work area. Creative places are co-constituted, each offering a unique landscape of affordances to the group. This collective conceptualization is defined by activity zones (e.g., behavior settings) within a building and considers how space is shaped by routine activities, processes, and use (i.e., affordances actualized) – as well as expanded by social activities and interactions. For example, a collaboration area, as a creative place, is defined by its architectural and interior design (spatial layout, furnishings and finishes, etc.) as well as patterns of social behavior. Examining these together could yield new insights, such as how architectural or interior designs sustain or inhibit creative behaviors, support organizational learning and culture, as well what conditions foster stability versus disruption (positive or negative) to the eco-psychological system. This conceptualization addresses Heft’s (2024) recommendation for greater consideration of the physical features and qualities of behavior setting. It also responds to his suggestion that the experiences of the individual be included by acknowledging that creative spaces are nested within creative places.

*Creative Environment (as Designed)* describes the creative work environment as conceived (e.g., explicitly designed and systematized) by architects, interior designers, planners, and others. It provides an ideological perspective, beginning with infrastructure (e.g., real estate, buildings, furnishings, fixtures, equipment, technologies, etc.) that organizes its intended use. For example, an AFO is designed with respect to the architectural and interior design layout, how these communicate intended use (such as through environmental graphics) as well as office policies (such as to discourage individuals from claiming the same work area day after day). This creative environment initially exists independent of users, defining the structures, features, and qualities of the work environment, including intended signifiers of environmental affordances. The environment is dynamic; it may be reconfigured by expansion or contraction of the physical infrastructure, adoption of new technologies, adjustments to operational policies, etc. Similar, conceptually, *to conceived space*, it could be considered an example of extended cognition – the design itself manifesting ideological and normative concepts that underlie theories of creativity and innovation as well as how space is systematized, technologically pre-defined, modeled, calculated, and/or structured for use.

Together spaces, places, and environments might best be conceptualized as a dynamic and nested ecosystem. This extends Lefebvre’s (1991) concept of “produced” space to integrate contributions grounded in affordance and behavior setting theory, acknowledging the action-oriented operationalization of creativity as embodied, embedded, enacted, and extended in designed environments. The ecosystem conceptualization provides a way to determine what is “inside” versus “outside” the system as a functional unit of analysis. However, ecosystems are not isolated, they influence and are influenced by other ecosystems and events “outside” the ecosystem. For example, the Covid-19 pandemic, in this conceptualization, would be considered an event external to the ecosystem that impacted it in different ways including the environment (e.g., changes in policies and infrastructure to protect user health), places (e.g., social distancing practices), and spaces (e.g., remote or hybrid modalities). These influences from outside the ecosystem exist in the larger ecosphere of influence.

## Conclusion and limitations

In conclusion, Propositions 1–3 begin to suggest ways that future creativity research and placemaking design strategies might be shaped by a novel approach, grounded in ecological psychology and 4E cognition. From the design perspective, an embodied-enactive lens, which considers the dynamic interactions between body and architectural setting, may provide a bridge to neuroscientific and psychological evidence through its focus on sensorimotor coupling between person and environment, embodiment, and affordances, lending greater empirical support for design strategies beyond replication based on anecdotal evidence. From the research perspective, it is (perhaps aspirational) that a 4E approach will help shape a change in “epistemological position, inviting creativity researchers to consider methods that account for person-environment coupling, the role of action in creative cognition, and examination of “creativity-in-the-wild” – in other words, from a dynamical systems approach in real-world settings.

This conceptual analysis is limited to western perspectives of creativity and environmental design. It also considers creative placemaking from an architectural design perspective, focusing mainly on commercial (e.g., office designs) and academic sectors because these have more often been the focus of efforts to utilize environmental design to enhance user creativity. Additionally, the analysis did not entail efforts to collect research related to remote or hybrid creative work. Including other perspectives might surface new or different barriers to empirically examining and designing for person-environment interactions that support creative endeavors. Finally, this conceptual analysis is based on a relatively small and methodologically diverse body of research and is grounded in the rapidly evolving field of 4E cognitive science. The propositions presented are not intended as prescriptive or the only approach to solving the identified problems. Instead, they might best be considered a “call to action” as a first step towards remedying several significant challenges, describing one possible path forward towards better understanding the role of the physical environment in creativity.

## Footnotes

1 Collectively, built environment disciplines.

2 Defined here as *consciousness/self-awareness*.

3 Despite this, there is a long history of environmental designs to foster creativity. E.g., Jefferson’s early American college incorporated views of nature, extensive lawns or open vistas, and integration of learning, living, and recreation facilities (Turner, 1984, p.3).

4 Distinguished by work involving complex problem solving and the need to come up with new products or services (Dul et al., 2011).

5 For example, Pixar and Google in California; Pier 57 and Etsy in New York; Lego in Billund, Denmark.

6 Amabile (1998) believed that physical space was less important than social factors and worthwhile considering as a factor in creativity.

7 Blomberg and Kallio (2022) posit five conceptualizations, from space considered fixed and existing separately from users to space constituted by people’s interactions or as a relational property of users.
